# Child-like sex dolls: legal, empirical, and ethical perspectives

**DOI:** 10.1038/s41443-024-00979-3

**Published:** 2024-09-28

**Authors:** Jeanne C. Desbuleux, Johannes Fuss

**Affiliations:** Institute of Forensic Psychiatry and Sex Research, Center for Translational Neuro- and Behavioral Sciences, University of Duisburg-Essen, Essen, Germany

**Keywords:** Translational research, Risk factors

## Abstract

The review deals with the controversy surrounding the use of highly realistic dolls with a child-like appearance. It summarizes recent empirical findings and provides an overview of the different legal and ethical perspectives on this issue. Countries use different legal approaches to regulate the use or sale of child-like sex dolls. Although a causal link is assumed by some legislators between the prohibition of such dolls and the protection of children from sexual abuse, empirical studies do not support this causality. The imposition of bans will hinder empirical research on the potential use of alternative sexual outputs for people with paraphilic disorders.

So called *child-like sex dolls*, mimicking the bodies of minors, have sparked widespread controversy, extending beyond media exposure to political and scientific discourse. This heightened attention partly stems from instances where such dolls were discovered with sexual offenders or were temporarily sold by major online retailers [1]. In some countries, the issue has gained prominence following legal reforms that criminalize the possession and distribution of child-like sex dolls.

In this paper, we aim to present various theoretical and moral perspectives, review the scarce empirical data, outline the moral-ethical and legal discussions, and provide an overview of the current situation and future prospects.

## Overview and definition

For clarity, we shortly define key terms relevant to this paper: *Sex dolls* are defined as highly realistic, anatomically accurate figures, mainly used for sexual stimulation or satisfaction [2], while *sex robots* are additionally equipped with artificial intelligence or technical extras (e.g., they can turn their heads or speak simple sentences). Dolls or robots differ from other sex toys in their complete replica of a human body although it is also possible to only buy a part of a body, like a torso. However, the early inflatable versions have considerably evolved since then and today’s dolls are tailored to customer preferences, raising debates about their classification as mere sex toys or as non-human partners.

In addition to sexual purposes, dolls, and robots also serve a number of non-sexual needs, such as the “physical” presence of a person, for cuddling or simply as a model that can be dressed according to user’s wishes [3–5]. While high-quality adult dolls are easily accessible online and customizable, sex robots are still in nascent stages of development. The costs for these dolls, with or without artificial intelligence, range from $1000 to $10,000, varying based on factors like body structure, additional features such as human hair, and materials like silicone or thermoplastic elastomers.

*Child-like sex dolls* specifically mimic the shape and size of real children’s bodies. They differ from so called *reborn dolls* in that they have body openings that can be used for sexually penetrative acts. By definition, reborn dolls are more seen as a work of art that represents the perfect replication of a human newborn or toddler. The legal definition of what constitutes a child-like sex doll, however, is unclear across jurisdictions, causing uncertainty among users about whether highly sexualized dolls with small stature but adult features (such as prominent breasts) fall under this category [6].

A *pedophilic disorder* involves an adult experiencing arousing fantasies or sexual desires for children, either acting on them or experiencing significant distress as a result [7]. *Pedo-/hebephilia* refers to a sexual interest for children or individuals with a prepubertal or peri-pubertal body type [8]. It is crucial to distinguish between pedophilia and child sexual abuse, as the public often conflates these terms. As academics repeatedly emphasize, having a pedophilic sexual interest is neither a prerequisite nor a guarantee for committing sexual offenses against children [9].

## Balancing moral concerns and potential benefits

The use of dolls, especially child-like sex dolls, is often the subject of polarizing discussions. Researchers [3, 10] have synthesized various perspectives and propose a framework for understanding the use of dolls, categorizing almost all arguments into three broad directions.

On a behavioral level, critics who see dolls merely as a *risk factor*, fear serious consequences from the use of them [11, 12], e.g., by lowering the inhibition threshold for (child) sexual abuse. For example, if an individual regularly interacts with a child-like sex robot programmed to simulate consent, it could lead to the erroneous belief that sexual arousal should always culminate in sexual activity [10]. Furthermore, it might reinforce the dangerous notion that children, perceived as sexual beings, can validly give consent.

Critics also emphasize the potential for these dolls to further objectify women and children [13, 14], potentially encouraging harmful behaviors associated with objectification [15, 16]. According to them, the use of adult female and child-like sex dolls leads to men acting out their sexual needs or their desire for power, creating a breeding ground for the degrading treatment of women and children as (sex) objects [13, 14]; also seeing dolls as the *materialization of sexual violence* against children [17]. Furthermore, it is assumed that men *train* aggressive behavior so that it is to be expected that this behavior will be transferred to interpersonal relationships in a second step. Interestingly, the assumed causal connection is often referenced in political decisions [18, 19] and proposed legislation, like the CREEPER Act in the United States (“The dolls and robots not only lead to rape, but they make rape easier by teaching the rapist about how to overcome resistance and subdue the victim.”; [18]), despite the fact that this hypothesis has neither been conclusively proven nor disproven by scientific research.

The use of child-like sex dolls is also discussed as a potentially “safe outlet” (see, for example, Michael Seto, quoted in a newspaper article; [20]), fitting into the view that dolls could be a *protective mechanism* [10]. Thus, dolls could be used by people with, e.g., a pedophilic sexual interest, to experience sexual satisfaction, thereby even reducing the risk of children becoming victims of sexual violence [10]. This idea might be supported by findings related to pornography consumption, where increased availability has correlated with decreased sexual violence at the population level in some Western countries ([3, 21, 22]; but see also [23] for a different view); similarly, the decrease in reported cases of child sexual abuse was associated with the legalization and accessibility of pornography in the Czech Republic [24].

Others [25] draw parallels between the use of robots or dolls and the use of computer-generated sexual depictions of children. Both involve fictional representations without a legal or moral victim, suggesting that they could offer an alternative for individuals with pedophilic sexual interests to live out their sexual fantasies without harming children.

Cantor [26] points out that masturbation is sometimes the only sexual outlet for people who have no opportunity for a romantic or sexual relationship. He considers the emotional consequences of such a limited sexuality. Especially for people with certain paraphilias, such as an exclusive pedophilic interest, access to lifelike dolls, to which Cantor refers as a “piece of latex” [13], could satisfy both sexual and emotional (e.g., emotional intimacy or companionship; [5, 27]) needs. Others also emphasize the possible positive effects that dolls could have, e.g., on lonely or traumatized people or on satisfaction and fulfillment in the general population [28, 29].

Lastly, regardless of whether the dolls have a risk-inducing or protective effect, they can be seen as a *functional tool* to enhance users’ life satisfaction by substituting for human interaction in various contexts [10]. They can act as partners for individuals who struggle to find a partner due to unrealistic expectations or limited social contacts [10]. Additionally, dolls provide a masturbation aid for those who cannot act out their fantasies with others. These functional uses of dolls address specific needs and can improve mental well-being, demonstrating their potential to fulfill important emotional and psychological roles.

## Moral considerations in philosophical debates

Another approach is offered by philosophical and sociological considerations, which, although not empirical, aim to establish a framework for the moral evaluation of right and wrong. Danaher [30, 31] advocates for an experimental approach to new technologies, emphasizing open exploration rather than immediate prohibition, particularly when empirical data are lacking. One argument supporting this approach are the potential therapeutic benefits for individuals with a pedophilic disorder. However, Danaher also acknowledges the counter-argument that certain behaviors, such as sexual acts with child-like robots, may be inherently immoral, warranting restrictions on moral grounds alone. He further argues that possession of child-like sex dolls and robots could indicate a lack of moral virtue, potentially leading to inclusion in registries for individuals with sexual convictions. Strikwerda [25] contends that child-like sex dolls and robots should be banned on moral grounds unless empirical evidence demonstrates they protect real children from harm. She argues that these behaviors undermine respect for others, as they lack equality and reciprocity, making them morally reprehensible from a legal moralism perspective. Chatterjee [12] highlights the potential cultural harm, suggesting that allowing trade in child-like sex dolls and robots could promote the depiction of children as sexual objects and normalize child abuse.

Harper and Lievesley [32] counter these arguments by emphasizing that, despite the lack of empirical data, some scientists may overestimate potential risks and underestimate potential therapeutic benefits when discussing the issue through a moral lens.

## Legal situation (in Western countries)

Various countries ban child-like sex dolls through a combination of existing and newly enacted laws, while adult-like dolls stay legal. In 2020, the Scientific Service of the German Bundestag wrote a summary of the legal regulation of child-like sex dolls in selected countries. It states that in the “majority of countries, certain uses of CLSD [child-like sex dolls] are probably regarded as punishable child pornography […] if certain images of them or of using them are produced, stored or distributed” [33]. A comparative analysis of these regulations [33–35] focuses primarily on Western nations and highlights significant differences. Below is a brief overview of the reasons cited by some selected countries to illustrate the diversity of their approaches. The ban on child-like sex dolls is generally supported by arguments that they contribute to the normalization of the sexual abuse of children and thus potentially act as a gateway for this behavior.

Australia, Germany, and Denmark have issued explicit bans. Australia amended its Criminal Code in 2019 to prohibit the possession, import, and export of child-like sex dolls and to classify them as child abuse material, punishable by up to 15 years of imprisonment (§ 273A.1, § 233BAB; [33]). In Germany, these dolls were banned in 2021 by the inclusion of § 1841 in the Criminal Code, and Denmark followed suit in 2022 with § 235a, which prohibits the possession and distribution of child-like sex dolls [33].

The United Kingdom [36] and Norway [34], on the other hand, use existing laws: In the UK, the authorities apply old customs laws and classify child-like sex dolls as “indecent or obscene articles”, which makes their import, distribution, and sale illegal, while possession remains legal. Interestingly, possessing a photo of such a doll could in fact count as an illegal act. Norway classifies these dolls as child pornographic material under § 311(1) of the Norwegian Penal Code, which criminalizes sexual depictions of children.

Also, Canadian law [33, 37] prohibits importing and selling any obscene material, which can include child-like sex dolls, under obscenity and child pornography laws.

South Korea has allowed the import of adult dolls (in 2022), after initially banning them. Although there were no explicit laws, customs had previously invoked a legal clause that prohibits the import of goods that “harm the country’s beautiful traditions and public morals”. Child-like sex dolls remain banned in South Korea, treating them in line with child pornography laws [38].

In the United States of America, there are currently no federal laws regulating the use of child-like sex dolls, although individual states have signed laws banning dolls. Although bans have been proposed, they have been criticized as unconstitutional and have not been passed. Reasons for not passing included that the definition of dolls was considered too vague so that adult dolls would also fall under the ban. It was also criticized that the empirical link between the use and the resulting abuse of a child was not sufficiently proven. Nevertheless, an attempt is being made with the “CREEPER ACT”, which is a legislative proposal in the United States aimed specifically at the problem of child-like sex dolls and robots. This bill aims to ban both the import and interstate commerce of such dolls and robots that are designed to resemble the bodies of children [18].

In summary, different jurisdictions justify the ban either with the protection of children from sexual abuse (I), the prevention of the normalization of the sexual abuse of children (II) or the protection of public morals (III; [34, 35]). Nevertheless, legislators face a dilemma. On one hand, they want to punish a “victimless crime” to prevent a potential indirect threat. On the other hand, they must consider that banning these dolls might be counterproductive, as the dolls could potentially reduce the risk of child sexual abuse [34, 35].

## Empirical data

Empirical research on sex doll usage, especially child-like dolls, is limited and currently, there are only a handful of studies that approach the topic empirically [3, 4, 6, 10, 39]. The main results of the limited empirical research is summarized below.

User demographics don’t significantly differ between those attracted to children (*pedo-/hebephilic users*) and those attracted to adults (*teleiophilic users*; [3, 39]). Studies consistently find that sex doll users are predominantly white, heterosexual, middle-aged, single men, usually employed and relatively affluent [3–5, 39]. Psychometrically, doll users don’t fundamentally differ from non-users in psychosexual functioning. Sex dolls serve various purposes for users, from sexual satisfaction to companionship and artistic inspiration. In empirical surveys, roughly half of the doll users indicate that they view their doll as a partner, while the rest see it purely as a sex toy [4, 39]. Those in a partnership with their doll report emotional attachment and improved mental health through doll use, some even preferring dolls over human partners.

An online survey showed that both teleiophilic and pedo-/hebephilic users reported reduced sexual behaviors (like masturbation and pornography consumption) since using dolls, with the latter group noting a greater reduction in compulsive sexual behaviors [39]. Pedo-/hebephilic users primarily turned to dolls due to unfulfillable atypical sexual interests with humans, occasionally stating a decrease in their sexual interest in real children after using dolls.

Harper & Lievesley [3] studied psychological and risk factors associated with the possession of child-like sex dolls. They found that individuals with pedo-/hebephilic interests had a significantly stronger desire (79.2%) for doll ownership compared to teleiophilic participants (20–40%). In a further comparison between pedo-/hebephilic people with and without dolls, the tendency or arousal towards hypothetical child sexual abuse was found to be lower in doll owners. This was true even after adjustment for higher self-reported sexual attraction to children and objectifying behavior. There were no differences in self-reported delinquency between the two groups. The study also showed that doll owners tended to exhibit more schizotypal and less antisocial personality traits, with schizotypal traits associated with social withdrawal and creativity [29, 40], and antisocial traits associated with disinhibition and distance in interpersonal relationships [41–43].

In a qualitative study [6] in which users of child-like sex dolls were asked about the effects of the ban of such dolls (most participants being affected by the German law), many reported stigmatization and negative psychological effects. The ban was seen as restricting their ability to fulfill their sexual and emotional needs. The lack of clarity in the definition of *sex doll* led to uncertainty as to whether their doll falls under the ban. Only a few stated that they had given up their doll. A minority also stated that their risk for sexual offenses towards children had increased since the new law had come into effect because they lost an alternative sexual outlet.

In conclusion, current data do not indicate that virtual partnership and sexuality with child-like sex dolls increase a risk for real-life child sexual abuse. However, these are cross-sectional findings, and long-term studies, particularly in therapeutic contexts, are needed to assess causal effects. The users of such dolls, in contrast, indicate that it is a safe outlet for their sexual arousal pattern that does not produce a victim in contrast to the use of sexual abuse images or videos or child sexual abuse.

## Complexities in regulating child-like sex dolls

The fact that various countries are trying to counter the risk of sexual abuse of children, which the lawmakers assume is increased by the use of dolls, with imprecise laws is causing uncertainty. For example, the distinction between *reborn babies*, which are considered works of art, and infant sex dolls is not clear. The presence of a penetrable hole is often cited as the primary distinguishing feature, suggesting that sexual activity and sexual abuse necessarily involves penetration. However, this view overlooks the complexity of sexual acts that can occur without penetration, so emphasizing the physical structure of the doll (i.e. the presence or absence of a hole) is somewhat limiting. Furthermore, the practical possibility of modifying a doll to include such a hole suggests that physical design alone cannot be a sufficient criterion for legal or moral judgements. Theoretically, a person can legally own a reborn baby and encounter a hefty prison sentence when the person adds a sexual device to this doll such as a penetrable hole that may legally transform the doll into a sex doll.

The private nature of interactions with these dolls further complicates regulation and enforcement. It is virtually impossible to monitor or verify what a person does with their doll in the privacy of their home, which raises questions about the effectiveness and enforceability of doll possession laws. Is it legal and not punishable when a person cuddles with their reborn baby? Does it become sexual once the person kisses this doll or is such behavior also legally acceptable? Where is the line between legally acceptable behavior and those behaviors that can bring people for many years into prison? This issue also intersects with gender and assumptions about the risk of sexual offenses. For example, if societal stereotypes imply that women, who are less likely to have a pedophilic sexual interest [44], can possess such dolls without suspicion, while men, particularly those classified as “pedophiles”, cannot, this creates a potential gender bias in legal and ethical considerations. The core of this debate revolves around the intentions and behaviors of doll owners. This is interesting in that users of sex dolls also give many other, non-sexual reasons for using sex dolls. However, taken these reflections into account, it challenges simplistic approaches to regulation and highlights the need for an open discussion about pedophilia, meaningful treatment options of pedophilic disorder, and the complex relationship between sexual offenses and paraphilias.

## The complexity of sexualized dolls on abuse prevention

As mentioned above, the two opposing views on the effects of sex dolls are often in great conflict (e.g., [3, 10, 11]) with each other, but it is often not recognized that the use of these objects may not have any significant or measurable effects. Still, from a moral perspective, it is noteworthy that female dolls for adults were not subject to similar bans as child-like dolls, despite their (presumably) higher sales volume and thus higher relevance when one would assume that they also increase the risk of sexual violence against women. This raises questions about the consistency of and reasons for such legal distinctions. While it is not appropriate to directly compare or equate violence against women and children, it is important to recognize that power is exercised and behaviors are potentially trained in both forms and predominantly by male users [45]. This is particularly important when one considers that many cases of child sexual abuse are not due to pedophilic sexual interest [44, 46]. This observation is consistent with the broader understanding of sexual abuse as an expression of control and dominance, neuropsychological functioning (e.g. restraining one’s impulses) or personality traits (e.g., an antisocial personality disorder) that goes beyond the simple framework of sexual gratification [42, 47]. These considerations underline the complexity of the issues surrounding sexualized dolls and their social impact.

The premise that pedophilic sexual interest is unchangeable leads to considerations of successful treatment with the aim to protect children while allowing individuals with a pedophilic sexual interest to lead fulfilling lives (implying sexual satisfaction). There are often references to existing effective therapies for people with pedophilic disorder; for example, Danaher refers to the German Darkfield Project as “best practice” [30]. This approach focuses on assisting individuals to appropriately recognize and assess their sexual desires and needs, to help them identify and manage situations that pose a risk to others, and to acquire strategies for preventing sexual offenses [48]. However, these therapy methods also lack convincing evidence and are criticized both regarding the therapy methods offered and their scientific evaluation [49]. König [49] refers to a study by Nentzl and Scherner [50] that followed a group that received the treatment for six years following therapy. The results were not as promising as expected: 85.7% of the participants stated that they had committed new or additional sexual offenses after therapy. Of these, 4.2% admitted to sexual abuse of children, while 95.8% used images of sexual abuse. Furthermore, König’s interpretation of the data suggests that all of the men who had previously admitted to sexual offenses continued this behavior after treatment. In addition, 11.1% of participants who had not previously offended became first-time users of child sexual abuse images. In conclusion, similar as for other studies on the effects of sexual offender treatment, there is a lack of comprehensive prospective control group studies to validate their crime prevention efficacy or exclude potential harm [49, 51]. Without claiming that the use of dolls can be safe or useful, these findings at least suggest that it would make sense to be open to the development of alternative treatment methods for prevention to ultimately protect children and women from (sexual) violence.

Exploring the use of dolls appears to be a worthwhile endeavor. As mentioned by some researchers, one potential approach to safely study the use of highly realistic sex dolls would be to embed them as a therapeutic tool within existing treatment programs like Dunkelfeld or the Healthy Sex Programme in the UK, similar to other forms of fictional sexual materials such as written stories [52, 53]. Following the authors, this would allow for a controlled environment to test for potential positive or negative effects on sexual thoughts or behaviors [52]. This aligns with harm reduction principles, as seen in drug addiction treatment, which aims to minimize harm when eradication isn’t feasible [53–55]. Societal moral disgust often obstructs such approaches, prompting the question of whether legislation should be based on disgust. The key issue is whether current laws focus on societal repulsion towards pedophilic fantasies or genuinely aim to protect children. However, implementing such an approach would necessitate rigorous supervision and monitoring of participants, as well as extensive multidimensional research. Furthermore, significant legal challenges would need to be addressed, including securing a special license for the possession of these dolls.

The situation of individuals with pedophilic sexual interests or disorders should not be overlooked, even if it may seem less important to some people compared to the consequences of child sexual abuse. Still, ignoring the situation is problematic for two main reasons: firstly, from a human rights perspective, and secondly, because stigmatization can lead to increased social isolation, which in turn can limit the ability to control behavior [56]. Additionally, people with pedo-/hebephilia are often confronted with aggression and rejection [49, 56, 57]. This may be partly due to the frequent incorrect equation of pedophilia and child sexual abuse.

## Limitations and conclusion

A limiting factor is that whenever scientists discuss the topic of child-like sex dolls and refer to empirical data on the one hand and demand empirical data on the other, they are dealing with self-reported data. These have advantages, such as providing direct insight into users’ perceptions, ease of implementation, and addressing a broad range of topics. However, it also has significant disadvantages, particularly in collecting data on the possession of child-like dolls, a criminal offense in many countries, leading to potential reporting bias to avoid legal consequences. Users may also bias responses due to social desirability or to avoid stereotypes. Larger clinical studies with various methods and long-term studies could enrich current knowledge.

It is likely that the demand for highly realistic dolls and robots will continue to rise. Not only because of technological progress but also because social acceptance of the use of humanoid robots is likely to increase as they become more common. As seen, the expected effects of the use of (child-like) sex dolls are often presented as immense and underpinned with drastic examples and wording. Many professionals [3, 10, 13, 14, 26] with different backgrounds have already weighed these arguments, whereby no common understanding is expected due to missing empirical research. Unlike other forms of sexually stimulating material, child-like dolls are inherently fictional. They are not sexualized objects by default; instead, their sexual nature emerges from the user’s imagination and actions. These dolls can be seen as fantasies brought to life, making them visible and potentially morally offensive to the public. It seems that the bans against dolls are not just about protecting children, but also about the desire for moral control, in which complete abstinence from any sexual behavior seems to be the only legitimate way of dealing with a pedophilic interest.

As Frommel [58] observes, current prevention strategies appear to rely predominantly on criminal law. She argues that this marks a new phase in anti-liberal criminal policy, where the distinction between law and morality is increasingly being eroded. This shift, she contends, essentially seeks to elevate the threat of punishment as an expression of state-imposed moral policy.
